# Development of dual-targeted nano-dandelion based on an oligomeric hyaluronic acid polymer targeting tumor-associated macrophages for combination therapy of non-small cell lung cancer

**DOI:** 10.1080/10717544.2019.1693707

**Published:** 2019-11-28

**Authors:** Bingjie Wang, Wei Zhang, Xiudi Zhou, Mengna Liu, Xiaoya Hou, Ziting Cheng, Daquan Chen

**Affiliations:** a School of Pharmacy, Key Laboratory of Molecular Pharmacology and Drug Evaluation (Yantai University), Ministry of Education, Collaborative Innovation Center of Advanced Drug Delivery System and Biotech Drugs, Universities of Shandong, Yantai University, Yantai, PR China;; b Department of Radiotherapy, Affiliated Yantai Yuhuangding Hospital of Qingdao University, Yantai, PR China;; c Department of Pharmacy, Binzhou People’s Hospital, Binzhou, PR China

**Keywords:** Combination therapy for non-small cell lung cancer, curcumin and baicalin co-delivery, dual-targeted nano-dandelion, oligomeric hyaluronic acid polymer, targeting tumor-associated macrophage

## Abstract

In this study, the novel carrier materials were screened to structure targeting nano-micelles (named ‘nano-dandelion’) for synchronous delivery of curcumin (Cur) and baicalin (Bai), which could effectively overcome the tumor resistance. Mannose (Man) was found to bind better to CD206 receptors on the surface of tumor-associated macrophages (TAMs), thereby increasing the number of nano-dandelion engulfed by TAMs. Furthermore, oligomeric hyaluronic acid (oHA) was able to target CD44 receptors, resulting in recruitment of a higher number of nano-dandelion to locate and engulf tumor cells. The disulfide bond (S–S) in 3,3′-dithiodipropionic acid (DA) could be broken by the high concentration of glutathione (GSH) in the tumor microenvironment (TME). Based on this, we selected DA to connect hydrophobic fragments (quercetin, Que) and oHA. A reduction-sensitive amphiphilic carrier material, quercetin–dithiodipropionic acid–oligomeric hyaluronic acid–mannose–ferulic acid (Que–S–S–oHA–Man–FA; QHMF) was fabricated and synthesized by ^1^H NMR. Next, QHMF self-assembled into nano-dandelion, i.e. encapsulated Cur and Bai in water. Critical experimental conditions in the preparation process of nano-dandelion that could affect its final properties were explored. Nano-dandelion with a small particle size (121.0 ± 15 nm) and good normal distribution (PI = 0.129) could easily enter tumor tissue through vascular barrier. In addition, nano-dandelion with a suitable surface potential (–20.33 ± 4.02 mV) could remain stable for a long duration. Furthermore, good cellular penetration and tumor cytotoxicity of nano-dandelion were demonstrated through *in vitro* cellular studies. Finally, effective antitumor activity and reduced side effects were confirmed through *in vivo* antitumor experiments in A549 tumor-bearing nude mice.

## 1. Introduction

As the second leading cause of death globally, cancer is a serious threat to human health (Rehman et al., 2018). Among all cancers, non-small cell lung cancer (NSCLC) is associated with a higher morbidity and mortality and has a poor prognosis (Hsiao et al., 2013; Jiang et al., 2019). Approximately, 75% of patients with NSCLC are diagnosed at an advanced stage due to lack of early screening and diagnosis. Furthermore, NSCLC is one of the most invasive and metastatic cancers, and the 5-year overall survival rate is very low. Subtypes of NSCLC include squamous cell carcinoma, large cell carcinoma, and adenocarcinoma (Zarogoulidis et al., 2013).

The current therapeutic strategies for NSCLC do not prove to be useful. For example, use of surgery in patients with early-stage disease has been established, but it may not completely cure or effectively inhibit the development of NSCLC. In addition to surgery, chemotherapy and radiotherapy are limited by their side effects, such as alopecia, vomiting, and other adverse reactions. Although many drugs are used to treat patients with NSCLC, such as cisplatin, their clinical application is limited by issues that need to be urgently addressed. These include low bioavailability, high toxicity, and associated side effects. The lack of selectivity is the biggest challenge associated with chemotherapy, leading to increased toxicity and drug resistance (Hayes & Wolf, 1990). Increasing selective accumulation of drugs at tumor sites could markedly reduce occurrences of toxicity and side effects associated with chemotherapy drugs. Targeted drug delivery is a strategy used to improve drug selectivity (Handali et al., 2018; Liu et al., 2018a,b; Herea et al., 2019). Therefore, a dual-targeted nano-dandelion-like antitumor therapeutic strategy was designed for NSCLC.

The in-depth study of the tumor microenvironment (TME) has identified that there were multiple differences between tumors and normal cells. For example, lower pH (Chen et al., 2014; Lv et al., 2016; Hong et al., 2017) and higher glutathione (GSH) levels (Cheng et al., 2011; Raza et al., 2018) have been reported in TME. Studies on tumor surface proteins have reported the presence of multiple specific receptors, such as CD44 receptors (Chen et al., 2016, 2017a; Dong et al., 2018), folate receptors (Chen et al., 2017b), glycyrrhetinic acid receptors (Tian et al., 2012; Sun et al., 2017; Wang et al., 2018b; Yan et al., 2018), and transferrin receptors (Zhao et al., 2018). Based on the specificities of tumor cells, formulation workers have designed a range of targeted agents for the targeted delivery of anti-cancer drugs to tumor sites (Wang et al., 2018a, 2018c).

Macrophages (M cells), one of the essential immune cells, are involved in the recognition, phagocytosis, and killing of damaged tissues and potentially malignant cells (Yang et al., 2019). M cells can respond to different environmental signals (Murray et al., 2014) and be classified as either M1-type (tumor-suppressive) or M2-type (tumor-promoting) (Ostuni et al., 2015; Ngambenjawong et al., 2017; Qiu et al., 2018; Räihä & Puolakkainen, 2018; Liu et al., 2019). Interestingly, both M1 type and M2 types retain the capacity for plasticity (Quail & Joyce, 2017; Yuan et al., 2017; Zhao et al., 2018). After reprograming by the TME, M cells are converted into M2-type and termed tumor-associated macrophages (TAMs). TAMs are key components of the TME that can contribute to tumor growth, reproduction, and metastasis (Ngambenjawong et al., 2017; Santoni et al., 2017; Singh et al., 2017; Farajzadeh Valilou et al., 2018; Hegab et al., 2018; Qiu et al., 2018; Räihä & Puolakkainen, 2018). The polarization from M2 type to M1 type can not only reduce the immunosuppressive constraints, but also augment the chemotherapy efficacy (Dexi et al., 2013; Igor & Yuri, 2015). It had been proved that there were a lot of special targeting ligands. Mannose receptor (CD206) was one of the most frequently targeted receptors overexpression on the surface of M2-TAMs (Saijie et al., 2013; Yang et al., 2018). Furthermore, transferrin receptor, legumain and folate receptor beta are also three other targeted receptors overexpressed on M2-TAMs (Wenyuan, 2006; Amaya, 2009; Gianfranca, 2010; Yoshiyuki et al., 2015). In this study, a nano-dandelion was designed, which could target M2-type cells to precisely kill M2-type cells or reprogram M2-type cells into M1-type cells. The literature search revealed that many researchers had investigated the transformation of TAMs considering various model drugs, such as chloroquine (Chen et al., 2018), baicalin (Bai) (Tan et al., 2015), and regorafenib (Zhao et al., 2018). In this study, Bai was selected as a model drug.

The biopolymer oligomeric hyaluronic acid (oHA) is used frequently as a biodegradable material in the field of nano-delivery systems because of its biocompatibility, high water solubility, and its ability to be easily chemically modified (Labie et al., 2019). In addition, oHA is specifically recognized by CD44 receptors (Jiang et al., 2014; Assanhou et al., 2015). In our study, oHA was used as a hydrophilic targeting material. 3,3′-Dithiodipropionic acid (DA) containing a disulfide bond (S–S) was used as the truss arm. The S–S could be reduced and destroyed by high concentration of GSH in the TME (Raina & Missiakas, 1997; Suhaas et al., 2009; Chen et al., 2017a; Wang et al., 2018a). Mannose (Man) had been reported that it could bind better to CD206 receptors on the surface of TAMs (Yang et al., 2018). So, Man was selected to modify nanomaterials, which could target the CD206 receptors of TAMs. Quercetin (Que) and ferulic acid (FA), two natural active monomers used in traditional Chinese medicine, have broad biological activity, including expectorant, anti-tussive, lipid lowering, and anti-cancer, which were applied to improve the hydrophobicity of carrier materials.

Curcumin (Cur) has broad biological activity, including anti-inflammatory, antitumor, and anti-oxidation. Hence, Cur has been used by several researchers as an antitumor drug (Abdallah et al., 2018; Zhang et al., 2018; Barati et al., 2019; Kundur et al., 2019). Compared with other antitumor drugs, Cur is associated with fewer side effects. Therefore, Cur was selected as an antitumor drug and combined with Bai to enhance the antitumor effects.

**Figure 1.**
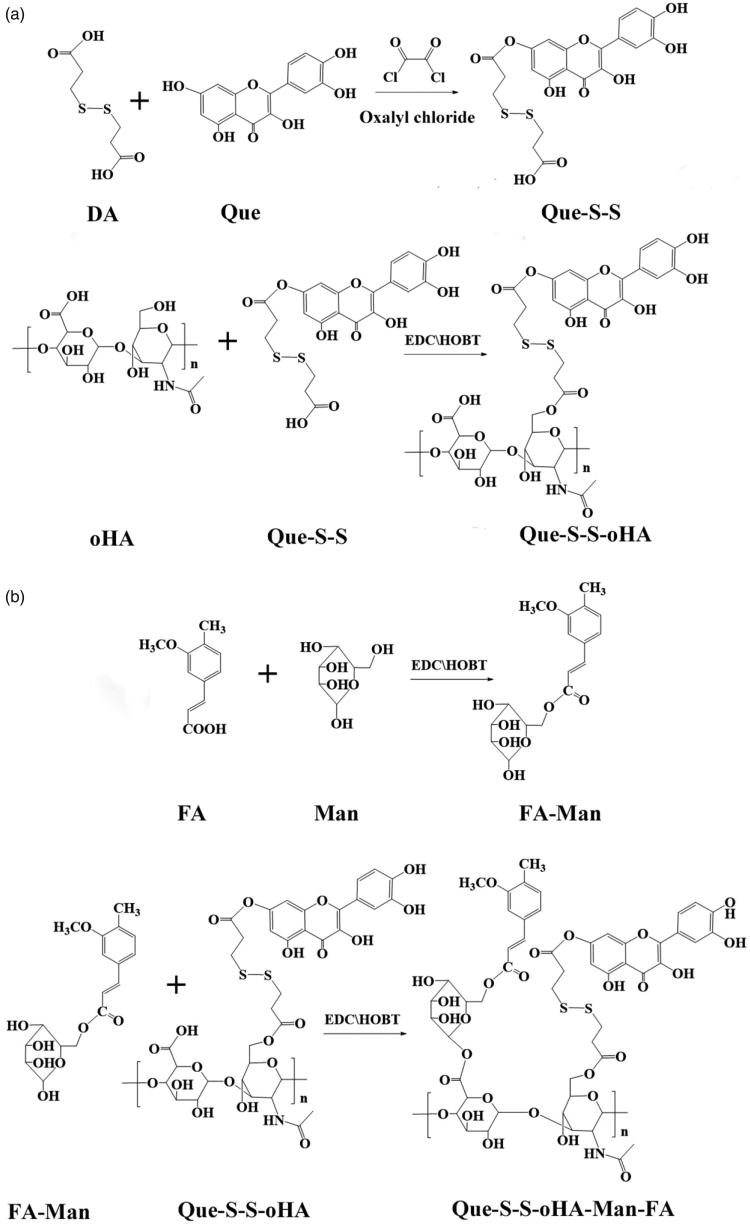
Synthesis of QH (a) and QHMF (a, b).

In this study, quercetin–dithiodipropionic acid–oligomeric hyaluronic acid–mannose–ferulic acid (Que–S–S–oHA–Man–FA, QHMF) (Figure 1) was successfully synthesized to prepare Man/oHA-based nano-dandelion-like nano-micelles (NMs) for codelivery of Cur and Bai (Cur/Bai) targeting both A549 cells and TAMs. Specifically, on the one hand, nano-dandelion with oHA targeting CD44 receptors could facilitate uptake of nano-dandelion in tumor locations. On the other hand, nano-dandelion with Man could be easily engulfed by TAMs via specific binding of Man to CD206. S–S was used as truss arm to combine hydrophobic and hydrophilic parts, facilitating the release of Cur/Bai from nano-dandelion in the TME. Notably, an advantage of the nano-dandelion was synergistic treatment (combined chemotherapy and immunotherapy).

**Figure 2.**
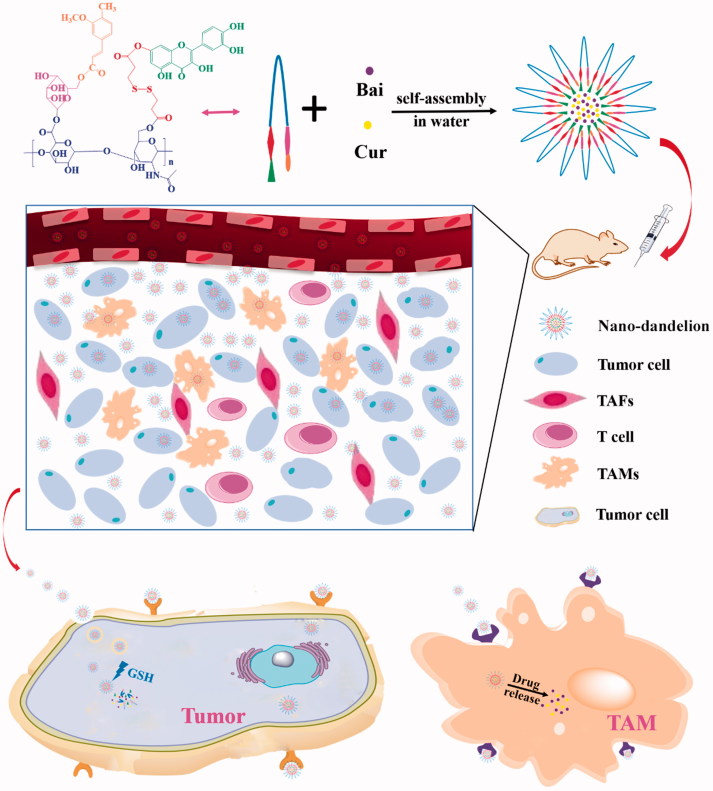
Schematic representation of QHMF self-assembly into nano-dandelion and the nano-dandelion targeting A549 cells and TAMs.

As shown in Figure 2, the nano-dandelion was successfully designed to exert beneficial effects at tumor sites based on intelligent delivery characteristics. In our study, the nano-dandelion was successfully prepared, which underwent preliminary evaluations, including determination of particle size and zeta potential and transmission electron microscope (TEM, H-600; Hitachi, Tokyo, Japan) analysis. In addition, the nano-dandelion was also evaluated to determine *in vitro* release, cellular toxicity and uptake, TAM repolarization, *in vivo* distribution, and pharmacodynamics. The results suggested that combining therapy may be a promising therapeutic strategy for patients with NSCLC.

## 2. Materials and methods

### 2.1. Materials

oHA (molecular weight <10 kDa) was obtained from Shandong Freda Co. Ltd. (Linyi, China). Que, Man, DA, FA, Bai, formamide, tetrahydrofuran (THF), and 1-hydroxybenzotriazole hydrate (HOBT) were obtained from Aladdin Reagent Net (Shanghai, China). Dimethyl sulfoxide (DMSO) and petroleum ether were acquired from Tianjin Fuyu Chemical Industrial Corporation (Tianjin, China). GSH was purchased from Sigma-Aldrich (St. Louis, MO, USA). Dulbecco's minimum essential medium (DMEM) was obtained from Saiersi Biotechnology Co. Ltd. (Shanghai, China). Fetal bovine serum (FBS) was obtained from Zhejiang Tianhang Biotechnology Co. Ltd. (Huzhou, China).

### 2.2. Methods

#### 2.2.1. Fabrication of QHMF nanocomposite

The QHMF was synthesized in two parts: Que–S–S–oHA (QH) and Man-FA. The first part included the synthesis of QH. First, 126.40 mg (0.5 mM) of DA was dissolved in 5 mL of THF, and 120 µL (0.60 mM) of oxalyl chloride was added dropwise into the DA solution at 0 °C. Then, the solutions were mixed for 4 h (35 °C) under stirring and the solvent in the reaction solution was vaporized by rotary evaporators. A total of 153.33 mg (0.60 mM) Que was dissolved into 5 mL of THF, added to the reaction bottle, and the reaction was continued for 24 h at 42 °C under stirring. Finally, the Que–S–S was collected by rotary evaporator. The Que–S–S was dissolved in 3 mL of DMSO, and 1.2 equivalents of EDCI (115.02 mg, 0.60 mM), and 1.2 equivalents of HOBT (81.08 mg, 0.60 mM) were added and mixed at 45 °C under magnetic stirring. After 4 h, oHA (201.65 mg, 0.5 mM) was dissolved in formamide (3.0 mL) and added dropwise into the Que–S–S solution and incubated at 52 °C with magnetic stirring for 48 h. Finally, the solvent was dislodged from the resulting solutions by dialyzing for 24 h at room temperature. The QH was obtained by lyophilization.

FA–Man was synthesized via the EDCI/HOBT. FA (97.00 mg, 0.50 mM) was dissolved in formamide (3.5 mL) at 60 °C. Then, 1.2 equivalents of EDCI (115.02 mg, 0.60 mM) and 1.4 equivalents of HOBT (94.59 mg, 0.70 mM) were dissolved in the formamide solution of FA. Next, the solutions were mixed at 45 °C for 4 h with magnetic stirring. Then, 1.2 equivalents of Man (108.10 mg, 0.60 mM) were dissolved in formamide (2 mL) and then mixed into previous solutions. The mixtures were incubated at 55 °C for 36 h with magnetic stirring.

The final step was the synthesis of QHMF. DMSO (6 mL) was used to dissolve QH, 1.4 equivalents of EDCI, and 1.6 equivalents of HOBT. Then, the solution was mixed at 42 °C for 5 h under stirring. Meanwhile, the formamide solution of FA–Man was added to the previous reaction bottle. The mixtures were incubated at 52 °C for 2 days with stirring. QHMF was obtained by dialysis and lyophilization.

#### 2.2.2. Characterization of QHMF

^1^H NMR was used to verify the structure of QHMF. QHMF (10.00 mg) was thoroughly dissolved in 0.6 mL of D_2_O and 0.3 mL of DMSO-d_6_, and the chemical shifts of QHMF were detected by ^1^H NMR.

#### 2.2.3. Preparation of drug-loaded nano-dandelion

The nano-dandelion was prepared by the dialysis method. First, 10 mg of QH or QHMF was dissolved in 4 mL of formamide. Then, Cur (1.5 mg) and Bai (0.5 mg) were synchronously dissolved in formamide (2.0 mL). Next, two different solutions were mixed and transferred to a dialysis bag (2000 Da) and dialyzed in PBS (pH 7.4) for 12 h in the dark. Then, the unloaded Cur/Bai were removed from the residual solution by centrifuging at 2000 rpm for 10 min. The prepared nano-dandelion was obtained from the supernatant.

#### 2.2.4. Characterization of nano-dandelion

The particle size and zeta potential of nano-dandelion were measured by Delsa Nano C (Beckman Coulter, Indianapolis, IN, USA). TEM was used to determine the morphological characteristics of nano-dandelion.

Drug loading (DL) and entrapment efficiency (EE) were examined by HPLC (Agilent 1260GB12C, Santa Clara, CA, USA) (Wang et al., 2018b). A 1 mL sample of the prepared nano-dandelion was disrupted with methanol (MeOH) and diluted to the desired concentration. Syringe filter membranes (0.22 μm) were selected to remove the impurities from the MeOH solution and then analyzed by HPLC at 425 nm (Cur) and 278 nm (Que). The EE and DL of the nano-dandelion were calculated as follows:

$$\mathrm{EE}\;(100\%)\;=\frac{\text{weight}\;\text{of}\;\text{drug}s\;\text{in}\;\text{NMs}}{\text{weight}\;\text{of}\;\text{the}\;\text{initial}\;\text{drugs}}\times 100\%$$

$$\text{DL}\;(100\%)=\frac{\text{weight}\;\text{of}\;\text{drugs}\;\text{in}\;\text{NMs}\;}{\text{weight}\;\text{of}\;\;\text{NMs}\;}\times 100\%$$

#### 2.2.5. In vitro reduction-sensitive release assay of nano-dandelion

First, the prepared nano-dandelion was concentrated by ultrafiltration (Millipore, Billerica, MA, USA, Amicon-Ultra-15, MW, 100 kDa) and then transferred into multiple dialysis bags (3000 Da). These dialysis bags were immersed in centrifuge tubes containing 45 mL PBS (pH 7.4, containing 0.5% Tween 80) with different concentrations of GSH (0, 0.1, 1, and 10 M). Subsequently, the tubes were incubated in a shaking (100 r/min) water bath at 37 °C. Release medium was sampled (1 mL) and the same amount of PBS was simultaneously added at the specified time point. The amount of Cur/Bai released was determined by HPLC at 425/278 nm.

#### 2.2.6. Cell culture

As CD44 and CD206 receptors are highly expressed on A549 and RAW264.7 cells, respectively, these two cell lines were used to evaluate the cellular levels of nano-dandelion. Both cell lines were cultured in DMEM supplemented with 10% FBS at 37 °C, 5% CO_2_.

#### 2.2.7. In vitro cell uptake and localization

**Concentration-dependence experiment.** In this study, the fluorescence of Cur was used. A549 cells were cultured in 12-well plates for 12 h. Different concentrations (Cur concentration, 5, 10, 20, and 40 µg/mL) of free Cur, QH@Cur NMs, and QHMF@Cur NMs were added to the 12-well plates and incubated for 4 h. After washing and fixing, images were obtained with a fluorescent microscope (Nikon, Tokyo, Japan) to monitor the cellular uptake of various NMs. Simultaneously, a concentration-dependence study on RAW264.7 was performed according to the above method using free Cur, QH@Cur NMs, and QHMF@Cur NMs.

**Time-dependence experiment.** The time-dependence course of free Cur, QH@Cur NMs and QHMF@Cur NMs was determined in A549 or RAW264.7 cells. First, A549 (RAW264.7) cells were seeded in a 12-well plate. After 12 h, the cells were incubated with free Cur, QH@Cur NMs, and QHMF@Cur NMs (Cur concentration, 40 µg/mL) for the indicated time. After washing, cells were fixed with 4% paraformaldehyde for 15 min. Finally, images were obtained with a fluorescent microscope.

**Cell localization studies.** Using Cur as a fluorescent probe and hochest33342 as a nuclear dye, the cellular localization of QHMF@Cur was determined using A549 (RAW264.7) cells seeded in six-well plates. Then, the cells were incubated with DMEM containing QHMF@Cur (Cur concentration, 40 µg/mL). The medium was removed after 4 h. After fixing and washing, 2 mL of hochest33342 (10 µg/mL) was used to stain cell nuclei. Finally, images showing cellular localization were obtained using a fluorescent microscope.

#### 2.2.8. Cell cytotoxicity assays in vitro

In this study, we investigated the cytotoxicity of free Cur, free Bai, vacant NMs, and different NMs in A549 and RAW264.7 cells by MTT assay. Briefly, A549 and RAW264.7 (A549/RAW264.7) cells were simultaneously plated in 96-well plates and left for 12 h prior to treatment with the prepared free Cur, free Bai, vacant NMs, and different NMs.

After incubating for 24 or 48 h, 20 μL of MTT solution (5 mg/mL) was dripped into 96-well plates and cells were incubated for 4 h. Then, the supernatant was carefully replaced by 200 mL of DMSO. Finally, the absorbance was measured using a microplate reader (Thermo Fisher Scientific Co., Waltham, MA, USA) at 570 nm after 10 min of oscillation. Cell viability (CV, %) was calculated according to the following formula:

$$\mathrm{CV}\;(\%)\;=\;\frac{\text{OD}\;\text{of}\;\text{treated}\;-\;\text{OD}\;\text{of}\;\text{zero}}{\text{OD}\;\text{of}\;\text{control}\;-\;\text{OD}\;\text{of}\;\text{zero}}\times 100$$

#### 2.2.9. TAM repolarization assays in vitro

Conditioned culture medium was collected from A549 cells. RAW264.7 cells were cultured in 96-well plates with collected culture medium. Then, the culture medium was removed, and the cells were treated with different concentrations of QHMF@Bai for 24 h. After collection and centrifugation, the levels of interleukin (IL)-6 and tumor necrosis factor (TNF)-α in the medium were measured by ELISA.

#### 2.2.10. In vivo fluorescence imaging

*In vivo* fluorescence imaging studies were performed using A549 lung cancer-bearing mice. The mice were treated with 150 µL free DiR, QH@DiR, or QHMF@DiR via tail vein injection once the tumor had grown to an appropriate size. The distribution of fluorescence *in vivo* was monitored at set time points by In-Vivo FX Pro (Bruker, Fällanden, Switzerland). Twelve hours after injection of DiR, tumor tissue and the major organs were harvested from mice, and fluorescence intensity of each organ was monitored using In-Vivo FX Pro.

#### 2.2.11. In vivo antitumor activity and histological analysis

The antitumor activity of NMs *in vivo* was investigated using ectopic A549 lung cancer-bearing mice, which were randomly divided into six groups. The antitumor efficacy of saline (control), free Cur, free Cur/Bai, QHMF@Cur, QH@Cur/Bai, and QHMF@Cur/Bai was evaluated in lung cancer-bearing mice following administration of multiple doses equivalent to 1 µg/g (Cur:Bai, 3.75:1.25) via tail vein injection. The tumor volume, as a therapeutic index, and body weight of mice, as a measure of toxicity, were recorded at each time point. The change in tumor volume in mice was used as an indicator of therapeutic efficacy. Finally, the major organs and tumor should be collected and fixed in 4% paraformaldehyde solution immediately after the mice were humanely sacrificed. After paraffin-imbedding and slicing, the sections were stained with hematoxylin–eosin (H&E) dyes and images showing histopathological evaluation were obtained by using a fluorescent microscope.

The size of the tumor was determined using the followed equation:

$$\text{Tumor}\;\text{volume}\;\left({\text{mm}}^{3}\right)=\;\text{length}\;\times {\;\text{width}}^{2}\times 0.5$$

## 3. Results

### 3.1. Characterization of QHMF

**Figure 3.**
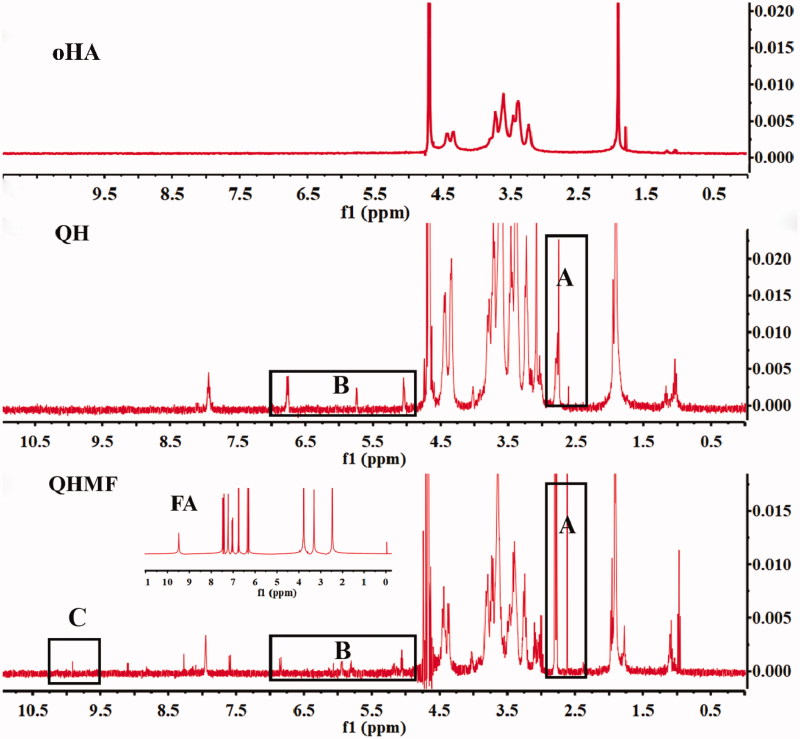
The ^1^H NMR spectra of QH and QHMF.

The ^1^H NMR spectra of QH and QHMF are shown in Figure 3. Compared with oHA, QH presented new chemical shifts located at approximately 2.6 and 2.8 ppm (Figure 3(A)) and 5–7 ppm (Figure 3(B)), which were typical signals of DA (CH_2_–S–S–CH_2_) and Que (the ‘H’ of benzene ring), respectively. Additionally, compared with QH, typical signal of FA was observed at 9.8 ppm (Figure 3(C)), which was the chemical shift of C=CH–COO. These results confirmed that QH and QHMF polymers were successfully obtained.

### 3.2. Characterization of QH NMs and QHMF NMs

**Figure 4.**
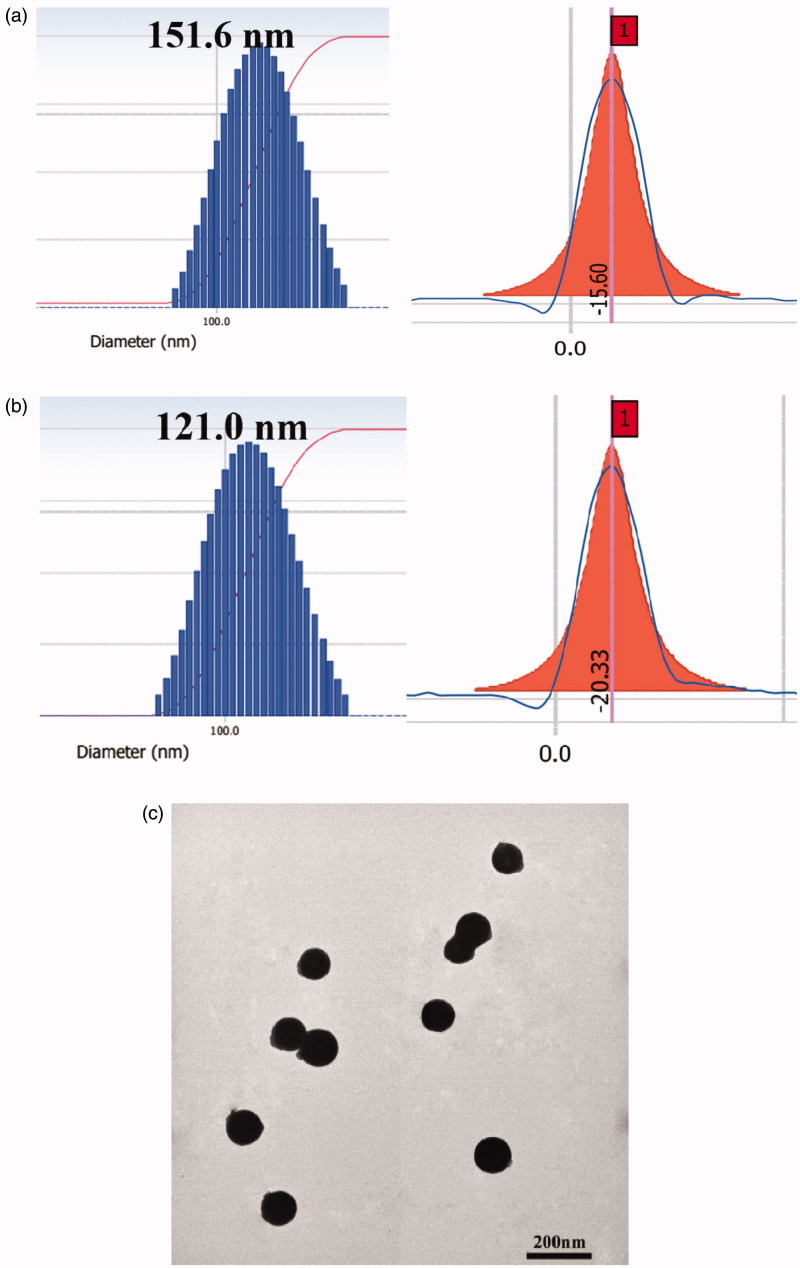
(a) The size and zeta potential of QH NMs. (b) The size and zeta potential of QHMF NMs. (c) TEM images of QHMF NMs.

The particle size, PI, zeta potential, EE% and DL% of QH NMs and QHMF NMs are illustrated in Table 1. As shown in Figure 4(a,b), QHMF NMs were smaller (121.0 ± 15 nm) than QH NMs (151.6 ± 12 nm). This may be attributed to the introduction of FA, which altered the hydrophobicity of QHMF and made QHMF NMs more compact. Furthermore, QHMF NMs presented a more stable zeta potential (–20.33 ± 4.02 mV), which could enhance the stability of QHMF NMs in systemic circulation.

**Table 1.** The physiochemical properties of different micelles.

	QH@Cur/Bai NMs		QHMF@Cur/Bai NMs	
Group	Cur	Bai	Cur	Bai
DL (%)	6.91 ± 1.38	3.26 ± 0.28	7.46 ± 1.70	3.50 ± 0.34
EE (%)	49.50 ± 9.3	67.32 ± 5.7	53.77 ± 11.5	72.63 ± 7.1
Size (nm)	151.6 ± 12		121.0 ± 15	
PI	0.139		0.129	
Zeta (mV)	–15.60 ± 3.47		–20.33 ± 4.02	

In addition, TEM images revealed that QHMF NMs were approximately spherical and uniformly distributed (Figure 4(c)). These results showed that QHMF NMs in a small spherical state were stable in water. Based on the above experimental results, QHMF was identified as a better carrier than QH. QH possessed one short hydrophobic segment. Conversely, the low rate of Que connection limited the hydrophobic properties of QH. The introduction of FA enabled QHMF to carry more hydrophobic segments, which increased the hydrophobic portion in the carrier material. Therefore, QHMF could easily self-assemble into micelles as a more compact structure in water.

### 3.3. *In vitro* reduction-sensitive releasing assay of NMs

**Figure 5.**
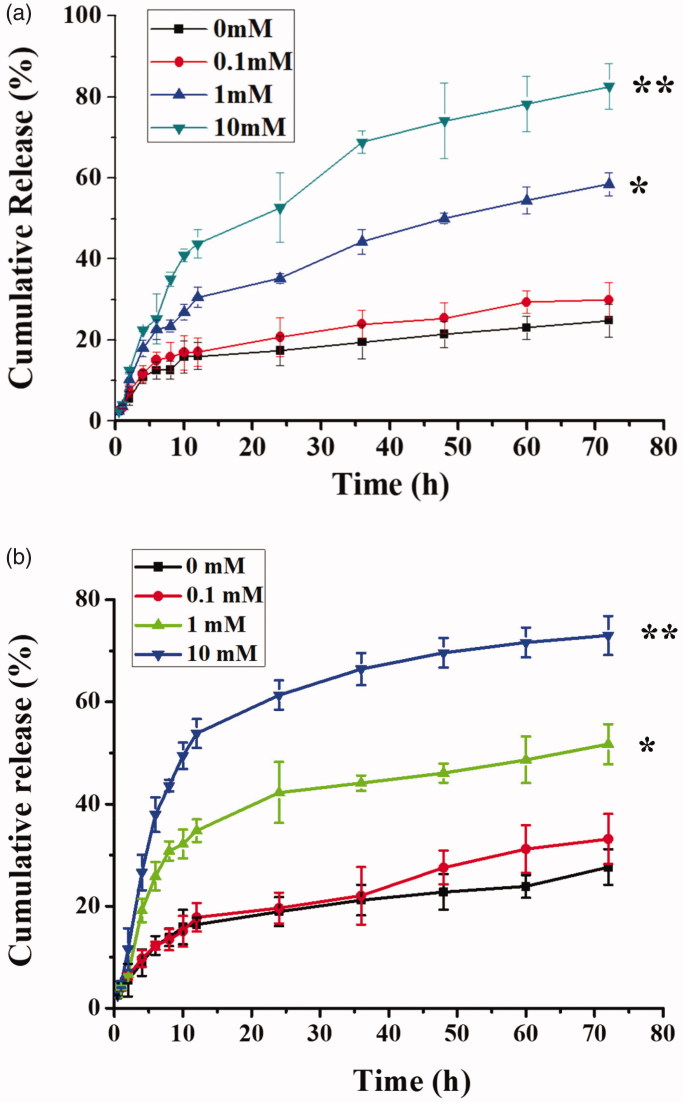
*In vitro* release profiles of Cur (a) and Bai (b) from the QHMF@Cur/Bai NMs in medium containing 0, 0.1, 1, and 10 mM of GSH.

A schematic showing Cur/Bai release by QHMF@Cur/Bai NMs is shown in Figure 5. There were significant differences in continuous drug release behavior and cumulative release of QHMF@Cur/Bai NMs in different release mediums (Figure 5).

The GSH-sensitive release of QHMF@Cur/Bai NMs was studied for three days. Previous studies confirmed that the size of QHMF@Cur/Bai NMs was stable for a few days. However, as shown in Figure 5, a large proportion of the drug was released on the first day. We speculated that the sustained release from micelles was poor. Compared to GSH 0 and GSH 0.1, GSH 1 and GSH 10, especially GSH 10, demonstrated higher cumulative release and a quicker release. In summary, the GSH-sensitivity of QHMF@Cur/Bai NMs was verified. These results indicate that the drug release of QHMF@Cur/Bai NMs was concentration (GSH) dependent. Thus, the drugs should be released quickly in the tumor cells (concentration of GSH, approximately 2–10 mM), providing sufficient concentrations of drug to kill tumor cells in the shortest amount of time.

### 3.4. *In vitro* cell uptake and localization

Selective uptake by A549 cells and RAW264.7 was the primary purpose of QHMF@Cur/Bai NMs. Moreover, selective uptake, which is an important factor affecting the final clinical effect of tumor chemotherapy, represents the greatest difference between targeted nano-delivery system and common delivery system. In this study, cellular uptake of different NMs was evaluated using an inverted fluorescence microscope (Figure 6). The uptake of drugs by both A549 and RAW264.7 cells markedly increased with prolonged administration time (Figure 6(a,c)) or increased drug concentration (Figure 6(b,d)). Thus, the uptake efficiency of QHMF NMs and QH NMs was time- and concentration-dependent in both cell types.

Figure 6.*In vitro* cellular uptake and localization. Time-dependent (a) and concentration-dependent (b) study in A549 cells; Time-dependent (c) and concentration-dependent study (d) in RAW264.7 cells. Localization study in A549 and RAW264.7 cells (e).

**Figure:**
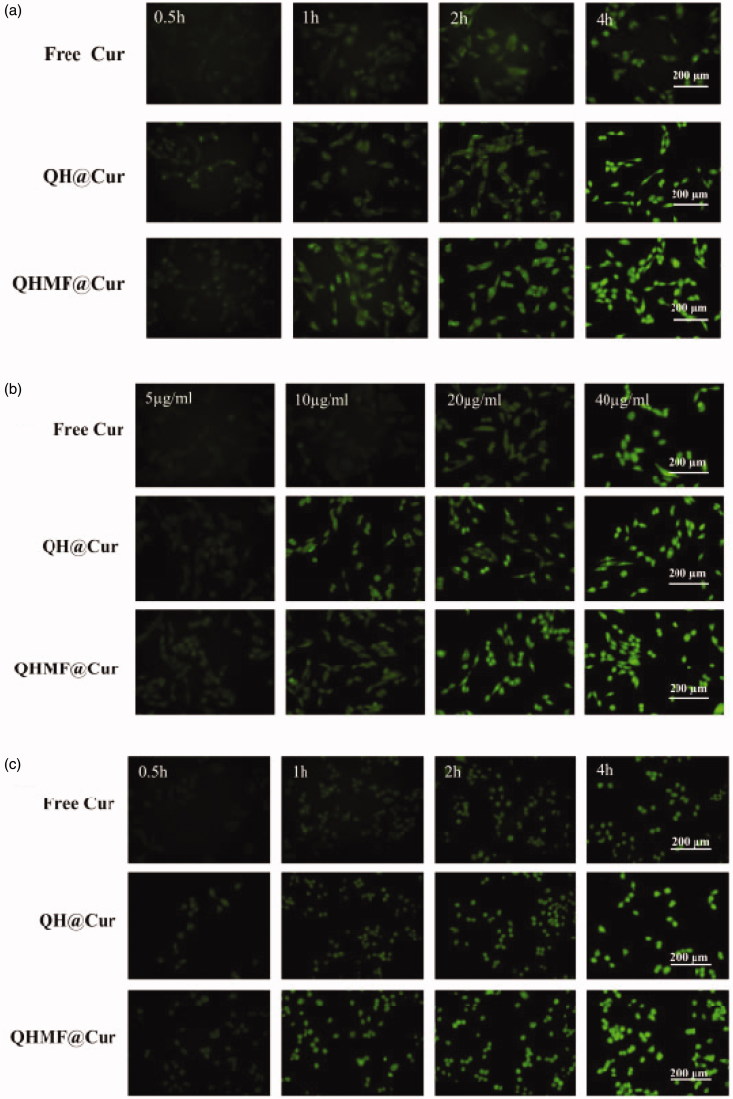

**Figure:**
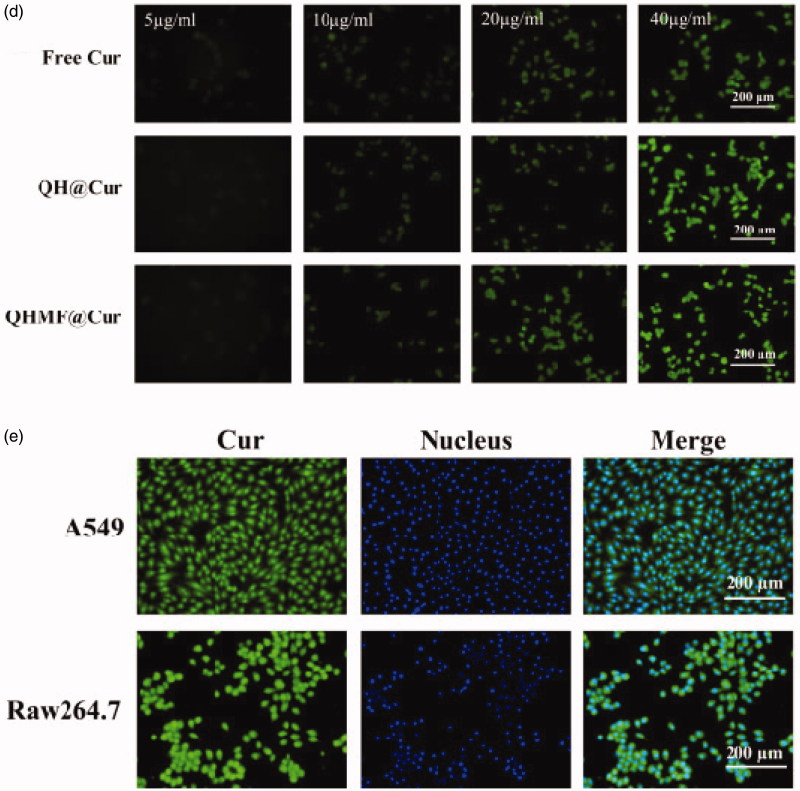

Figure 6(a–d) shows that the QHMF NMs are ingested more than the QH NMs by A549 and RAW264.7 cells under the same conditions. This was because the QHMF NMs had a higher EE than QH NMs. Furthermore, the QHMF NMs have a more compact structure and a smaller size than QH NMs, which allowed the QHMF NMs to more easily penetrate the membrane of the tumor cells (Desai et al., 1996). Additionally, QHMF NMs with Man could bind specifically to the CD206 receptor on the surface of TAMs, explaining why the QHMF NMs were ingested to a greater extent than the QH NMs by RAW264.7 cells (Alley et al., 2010; Harvey, 2011; Irannejad et al., 2013).

Furthermore, the QHMF NMs were mainly taken into the cytoplasm of A549 and RAW264.7 cells, as shown in Figure 6(e).

### 3.5. Cell cytotoxicity assays

**Figure 7.**
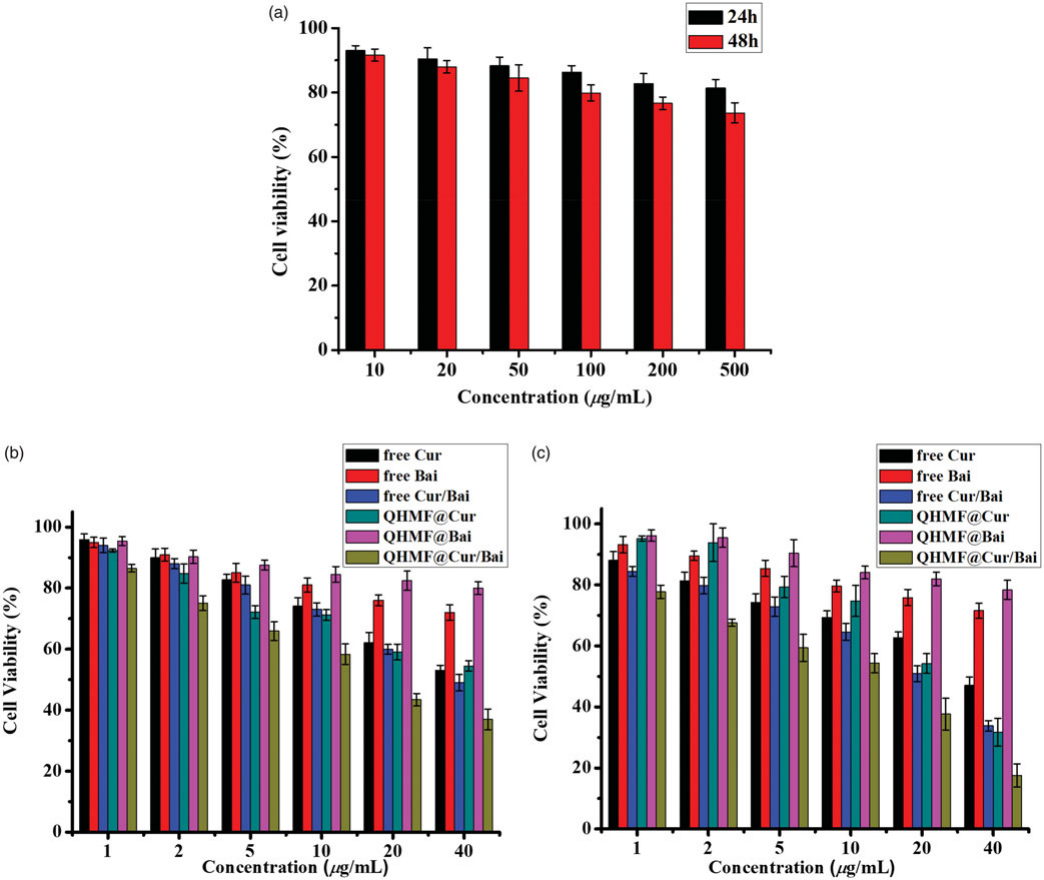
(a) Viability of A549 and RAW264.7 cells with QHMF at 24 and 48 h. The inhibiting effects of free Cur, free Bai, free Cur/Bai, QHMF@Cur, QHMF@Bai, and QHMF@Cur/Bai at 24 (b) and 48 h (c) on the A549 and RAW264.7 cells.

A549 cells overexpressing CD44 receptors and RAW264.7 cells overexpressing CD206 receptors were used in a cytotoxicity study. The inhibiting effects of free Cur, free Bai, free Cur/Bai, QHMF@Cur, QHMF@Bai, and QHMF@Cur/Bai on the A549/RAW264.7 cells were determined by the MTT assay (Figure 7).

Figure 7(a) shows that the carrier materials-QHMF induced little damage to A549 and RAW264.7 cells. The survival rate of cells remained above 70%, and was close to 80%, when the concentrations of carrier materials-QHMF reached 500 µg/mL following treatment for 48 h. The results proved that our synthetic carrier materials had low cytotoxicity. As shown in Figure 7(b,c), a clear reduction in cell viability was observed with increasing concentrations of drugs. Comparing with free drugs, it was found that the drug-loaded NMs had stronger inhibiting effects at the same treating time and the same concentration of drugs. In Figure 7(b,c), each figure showed that the combined treatment (Cur/Bai) was superior to monotherapy, Cur or Bai. Next, it was found Bai had less toxicities to A549 and RAW264.7 cells. However, when Bai was combined with Cur, a ‘1 + 1>2’ effect was observed, which indicated that Bai effectively enhanced the cellular inhibiting effects of Cur. In other words, a synergistic effect between Cur/Bai in tumor cells was accomplished by TAMs.

### 3.6. *In vitro* TAM repolarization assays

**Figure 8.**
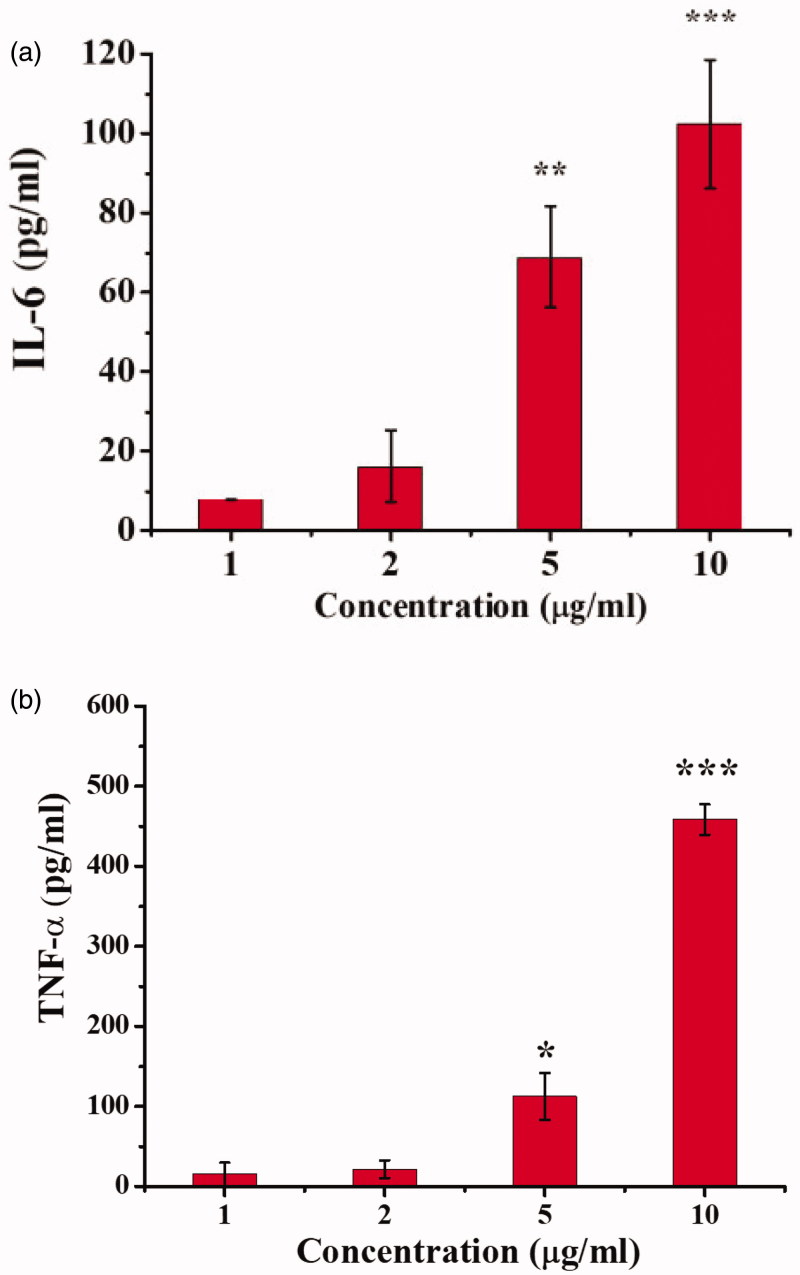
Interleukin-6 (a) and tumor necrosis factor-α (b) expression in TAMs after Bai treatment.

Bai had been reported to have the ability to reprogram TAMs toward an M1-like phenotype from M2-like phenotype. Next, to examine whether Bai could reprogram macrophages toward an M1-like phenotype, leading to tumor suppression, we measured the presence of IL-6 and TNF-α in the supernatant of RAW264.7 cells following treatment with Bai (Figure 8). High concentrations of Bai had been shown to inhibit the growth of RAW264.7. Figure 8 shows that amount of IL-6 (Figure 8(a)) and TNF-α (Figure 8(b)) secreted by RAW264.7 cells increased as the concentration of Bai increased. These results confirmed that Bai could reprogram TAMs. Thus, Bai had a promising role as an adjuvant chemotherapy at a lower dose.

### 3.7. *In vivo* real-time fluorescence imaging

**Figure 9.**
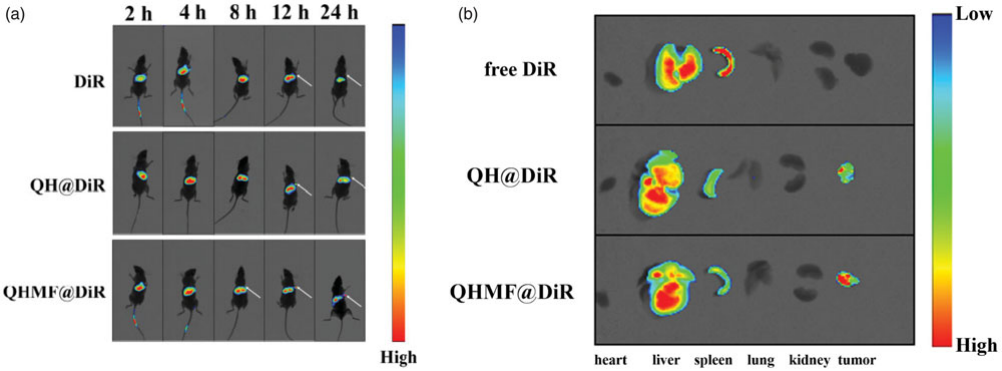
*In vivo* images of tumor-bearing mice. (a) *In vivo* fluorescence imaging of A549 tumor-bearing mice at different time points (2, 4, 8, 12, and 24 h) after intravenous injection of free DiR, QH@DiR, and QHMF@DiR. (b) Distribution of free DiR, QH@DiR, and QHMF@DiR in the heart, liver, spleen, lung, kidney, and tumor.

To estimate the biodistribution of QH NMs and QHMF NMs *in vivo*, DiR-labeled QH NMs and QHMF NMs were injected into A549 tumor-bearing nude mice, and *in vivo* real-time fluorescence imaging is shown in Figure 9(a).

Interestingly, QH@DiR NMs and QHMF@DiR NMs readily accumulated in tumors compared with free DiR, especially the QHMF@DiR NMs. Fluorescent imaging of isolated tissues (Figure 9(b)) showed that large amounts of DiR accumulated in the liver and spleen, because the liver is an important metabolic organ. After entering the circulatory system, the nanoparticles with a particle size of 50–200 nm were easily taken up by the reticuloendothelial system and accumulate in the liver (Lai & Guo, 2010; Chen et al., 2011; Huanan et al., 2013). In addition, as shown in Figure 9(a), DiR in the liver would be greatly reduced 24 h after administration.

### 3.8. *In vivo* antitumor activity

**Figure 10.**
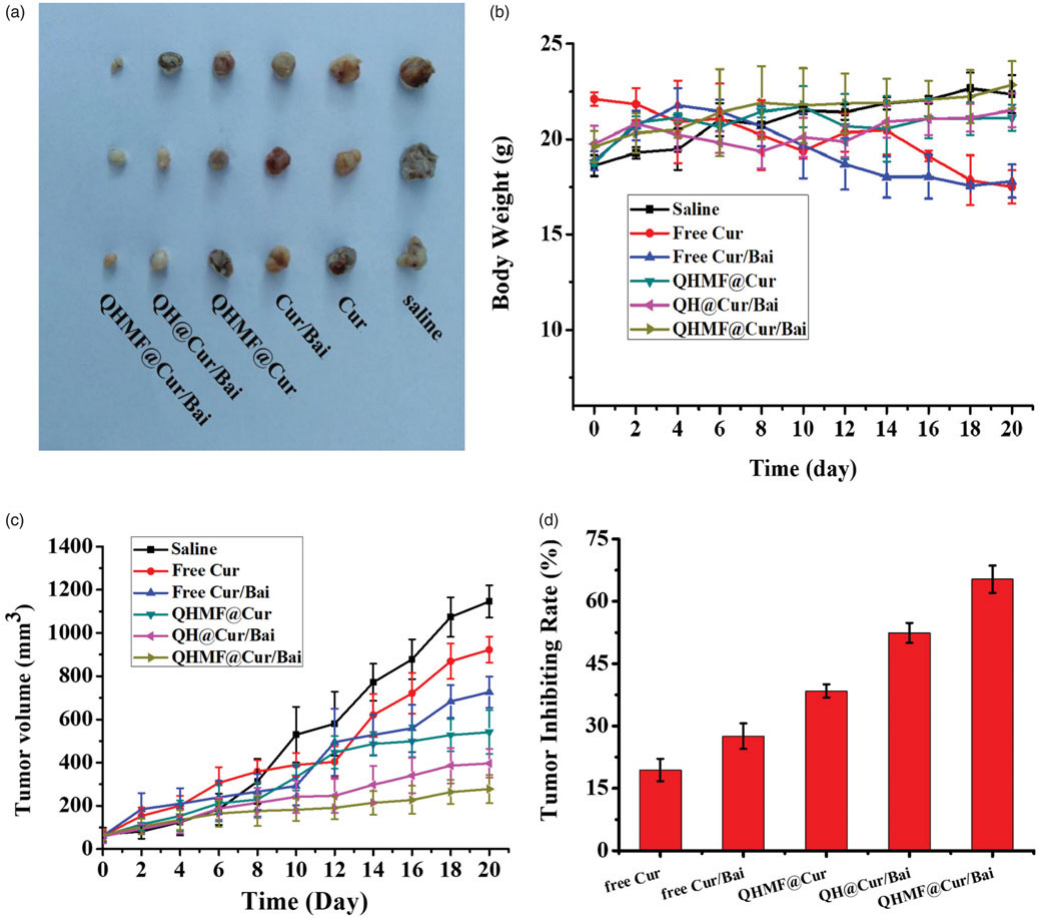
*In vivo* antitumor activity of NMs in tumor-bearing nude mice. (a) Tumor size. (b) Changes in the body weights of mice with time during treatment. (c) Changes in tumor volume over time. (d) The tumor inhibiting rate of different treated groups.

We evaluated the antitumor effects of saline (control), free Cur, free Cur/Bai, QHMF@Cur, QH@Cur/Bai, and QHMF@Cur/Bai (Figure 10).

First, changes in the body weights of mice are shown in Figure 10(b). No significant difference in body weight was observed between mice administered drugs and the control group. In contrast, there was a small loss of body weight in mice treated with free drugs compared with those treated with other formulations. There were some increases in body weight among the formulations treated. These results indicated that our formulation reduced the systemic toxicity caused by the free drugs. Figure 10(c) shows changes in the tumor volume of mice across the full dosing cycle, and Figure 10(a) shows the size of the tumor in nude mice in each group after the end of the treatment cycle. What is more, Figure 10(d) intuitively shows the tumor inhibiting rate of different treated groups. Results showed that the therapeutic effects of the different formulations were significantly better than those of the control groups. This might be explained by the preparation being more easily taken up and accumulated in the tumor tissue compared to free drugs. Interestingly, the tumor volume in QHMF@Cur/Bai-treated mice was smaller than that in QHMF@Cur-treated mice, which indicated that adjuvant treatment with Bai based on TAMs was effective. Furthermore, the rate of tumor inhibition with QHMF@Cur/Bai was greater than that with QH@Cur/Bai, which may be caused by QHMF@Cur/Bai with Man targeting the CD206 receptor of TAMs, and by QHMF@Cur/Bai having a more compact spatial structure, allowing it to be easily taken up by tumor cells.

### 3.9. Histological analysis

**Figure 11.**
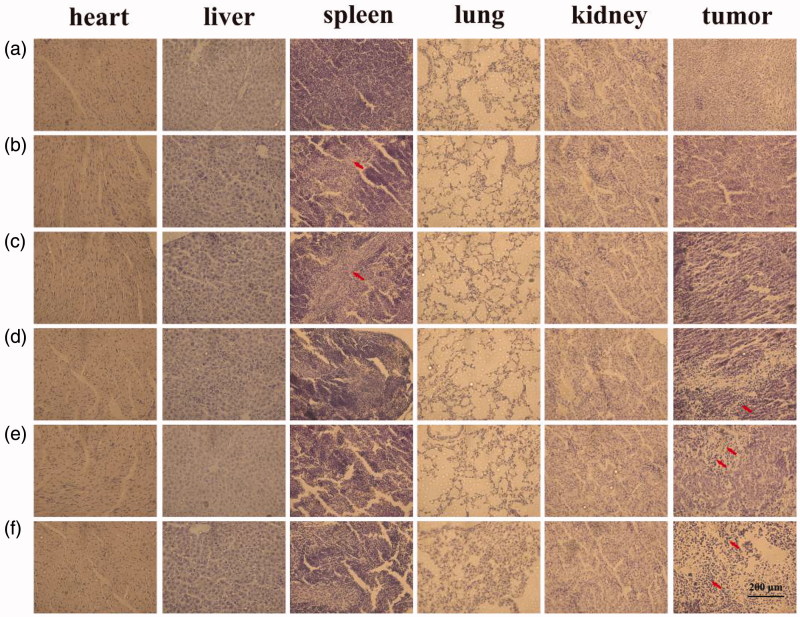
Histological images of tumors and major organs after different preparation processing. (a) Saline, (b) free Cur, (c) free Cur/Bai, (d) QHMF@Cur, (e) QH@Cur/Bai, and (f) QHMF@Cur/Bai.

The toxicity levels of various formulations were measured in tumors and major organs of mice. Histological analysis of tumor and major organs is shown in Figure 11. Free Cur and free Cur/Bai treatment showed damage to the liver. In contrast, a few or undetectable hepatic lesions was shown in the treated groups with QHMF@Cur, QH@Cur/Bai and QHMF@Cur/Bai, which testified that QHMF@Cur, QH@Cur/Bai, and QHMF@Cur/Bai had fewer physical toxicities compared with free Cur and free Cur/Bai. QHMF@Cur, QH@Cur/Bai, and QHMF@Cur/Bai treated groups show the good tumor inhibitory effects. As we can see from Figure 11, the tumor cells have obvious pyknosis, especially QHMF@Cur/Bai treated group.

## 4. Discussion

In this work, we demonstrated that Cur/Bai-based combination therapy had the better anti-cancer effects than individual Cur application, such as better A549 cells inhibition rate (*in vitro* cytotoxicity test) and tumor inhibition rate (*in vivo* pharmacodynamics experiment). Remodeling of TAMs had been successfully verified by detecting changes in the secretion of IL-6 and TNF-α secreted from TAMs and we realized that two sets of data were not abundant. We will study the specific proteins of M1 and M2 in the next experiment, which further proved the successful transformation of TAM. Besides, we found that baicalin could promote TAMs to secrete more IL-6 and TNF-α, but baicalein was not. It was not hard to speculate that β-D-glucuronide was an indispensable part for Bai when Bai plays a remodeling role. In the next following study, we will conduct more in-depth research, including the mechanism that Bai promotes the transformation of TAM, etc.

*In vivo* real-time fluorescence imaging also showed that the nano-preparations had better tumor targeting and aggregation. Although partial accumulation of nanoparticles in the liver and spleen is inevitable, we would further optimize the nanoparticles to reduce the accumulation in liver and spleen. In the next work, we will explore novel strategies based on TAMs transformation. We hope to develop strategies which can reduce the dose of chemotherapy drugs without reducing the efficacy. What is more, Dir fluorescence could be analyzed in the tail of mouse. This is because when we injected DiR into the tail vein of the mice, the operation was not skilled enough, which resulted in multiple injections (the same amount of injection). The DiR remains in the tail of the mouse and it was not cleaned up in time.

## 5. Conclusions

To further enhance the efficacy of Cur and to avoid development of multidrug resistance by tumor cells, we introduced Bai as an adjuvant therapy to reprogram TAMs. In this study, we designed a novel amphiphilic nanocarrier material (QHMF) with reduction-sensitive potential using few simple chemical reactions to self-assemble a micellar delivery system, nano-dandelion. We demonstrated that QHMF was successfully synthesized by ^1^H NMR. Analysis of particle size and morphology showed that nano-dandelion presented a uniformly distributed spherical structure of smaller size. Furthermore, nano-dandelion presented a suitable surface charge, which could be stable in systemic circulation. The reduction-sensitivity of nano-dandelion was demonstrated by an *in vitro* release assay. *In vitro* cell experiments showed that improved cellular uptake and cytotoxicity were achieved by nano-dandelion. Additionally, TAMs were successfully transformed into tumor-killing M1-type macrophages via reprograming by Bai, demonstrating promising role as a tumor adjuvant therapy. *In vivo* studies have shown that nano-dandelion could readily aggregate at tumor sites and exert more effective tumor-suppressive effect compared to free drugs. Furthermore, rate of tumor inhibition with QHMF@Cur/Bai NMs was higher than that with QHMF@Cur NMs, which further confirmed that Bai exerted an effective adjuvant antitumor effect. Overall, our proposed adjuvant tumor therapy based on reprograming of TAMs represents a promising strategy with good research prospects.
